# Development and Piezoelectric Properties of a Stack Units-Based Piezoelectric Device for Roadway Application

**DOI:** 10.3390/s21227708

**Published:** 2021-11-19

**Authors:** Chenchen Li, Fan Yang, Pengfei Liu, Chaoliang Fu, Quan Liu, Hongduo Zhao, Peng Lin

**Affiliations:** 1 The Key Laboratory of Road and Traffic Engineering, Ministry of Education, Tongji University, Shanghai 201804, China; lichenchen@tongji.edu.cn (C.L.); 15234058266@163.com (F.Y.); 2 Institute of Highway Engineering (ISAC), RWTH Aachen University, 52074 Aachen, Germany; liu@isac.rwth-aachen.de (P.L.); fu@isac.rwth-aachen.de (C.F.); q.liu@isac.rwth-aachen.de (Q.L.); 3 Civil Engineering and Geosciences, Delft University of Technology, 2628 CN Delft, The Netherlands

**Keywords:** pavement engineering, energy harvesting, piezoelectric device, stacked piezoelectric unit, piezoelectric properties, roadway application

## Abstract

To improve the energy harvesting efficiency of the piezoelectric device, a stack units-based structure was developed and verified. Factors such as stress distribution, load resistance, loads, and loading times influencing the piezoelectric properties were investigated using theoretical analysis and experimental tests. The results show that the unit number has a negative relationship with the generated energy and the stress distribution has no influence on the power generation of the piezoelectric unit array. However, with a small stress difference, units in a parallel connection can obtain high energy conversion efficiency. Additionally, loaded with the matched impedance of 275.0 kΩ at 10.0 kN and 10.0 Hz, the proposed device reached a maximum output power of 84.3 mW, which is enough to supply the low-power sensors. Moreover, the indoor load test illustrates that the electrical performance of the piezoelectric device was positively correlated with the simulated loads when loaded with matched resistance. Furthermore, the electrical property remained stable after the fatigue test of 100,000 cyclic loads. Subsequently, the field study confirmed that the developed piezoelectric device had novel piezoelectric properties with an open-circuit voltage of 190 V under an actual tire load, and the traffic parameters can be extracted from the voltage waveform.

## 1. Introduction

With increasingly prominent energy shortages and environmental pollution problems, the development and utilization of clean and renewable energy have been receiving increasing attention. Energy harvesting technologies, such as solar, geothermal, wind, and vibration energy harvesting, have been developing rapidly in recent years [1,2]. Among them, piezoelectric energy harvesters are extensively used in mechanical energy harvesting because of their high electrical conversion coefficient and stable structural performance [3]. If this technology can be utilized widely in road engineering, it could alleviate the current energy and environmental problems to a certain extent. In addition, piezoelectricity generated from traffic loads through these widely distributed piezoelectric devices could be applied to power electronics, such as signals, lights, and IoT systems, thus providing a new power solution for functional and intelligent roads [4,5].

The piezoelectric materials and structures significantly affect electricity conversion efficiency, structural strength, and durability [6]. Researchers have conducted theoretical analysis, numerical simulation, and experimental tests to investigate the mechanical and electrical performance of piezoelectric transducers. Varying kinds of structures are proposed, tested, and evaluated, such as cymbal type [7], bridge type [8], stacked type [9], and cantilever beam type [10]. The bridge and cymbal structures enhance the electrical properties by the angular amplification effect [11]. However, due to the stiffness difference between the piezoelectric ceramic and end-cap metal materials, the interface is prone to shear damage due to stress concentration, which seriously shortens the service life. Although the geometry and components of the cantilever structures can be optimized to resonate with the pavement, they still have the difficulty in mechanical resonance under the random impact of vehicle loads [12]. In contrast, the stack and pile structures have the advantages of high electromechanical conversion efficiency, high bearing capacity, and high durability, inducing a good prospect for pavement application [13,14]. Yang developed the stacked piezoelectric transducer and evaluated the factors’ influences on the electrical performance by a laboratory accelerated pavement testing system [9,15,16]. Wang proposed the optimal preparation process for the application of the stacked piezoelectric unit and investigated the electromechanical conversion performance and structural strength by indoor testing [17,18]. Li studied the electrical properties of piezoelectric units under different structural parameters, resistance, and traffic loads by laboratory tests, and given the attenuation law of piezoelectric properties under ultimate compression and cyclic loads [19].

Existing studies have shown that piezoelectric units installed in pavement suffer structural damages, such as interfacial shear failure, corner breaks, and electrode detachment [20,21]. Recent research has been shifted to assemble the piezoelectric transducers into arrays and fabricated them into a protective package to improve the energy conversion efficiency, structural strength, and service performance [22,23]. Roshani conducted uniaxial compression tests on piezoelectric devices and found that the number and arrangement of piezoelectric units influenced the output power [24]. Zhao proposed the use of a piezoelectric device based on an arch transducer array and discussed the synergistic performance between the device and asphalt pavement [25,26]. Jasim investigated the energy harvesting performance of a piezoelectric module in asphalt pavement through laboratory testing and multi-physics-based simulation [27]. Wang designed and assessed the stacked piezoelectric devices for pavements. In his research, the proposed device could harvest 11.67 mW at 0.7 MPa and 15 Hz with the corresponding optimum load of 10 kΩ [23]. In addition, Yang and Liu conducted experimental and simulation methods to optimize the structure of piezoelectric devices and analyzed the electrical and mechanical properties of the piezoelectric devices [22,28,29]. This research has provided useful references for the study of piezoelectric energy harvesters for roadway applications.

In summary, previous studies have conducted theoretical analysis, numerical simulation, and laboratory tests to promote the development of piezoelectric energy harvesting. However, the structural optimization design and efficiency improvement methods of the piezoelectric device are still in the exploratory stage and the influence of stress distribution, load resistance, vehicle load, and loading times on the electrical properties should be investigated further. Furthermore, the structural design and performance evaluation of the piezoelectric device still lacks on-site tests under actual road conditions.

To further improve the energy harvesting efficiency and compatibility in the pavement, this study proposed and fabricated a stack units-based piezoelectric device in the laboratory. To overcome the barriers of practical application, the influences of stress distribution, load resistance, vehicle load, and loading times on the piezoelectric performance were investigated using theoretical analysis and experimental testing. In addition, an on-site performance evaluation was also conducted to validate the actual piezoelectric properties in the actual road conditions. This study can further guide the optimization of the piezoelectric device structure and contribute to the application for roadways.

## 2. Piezoelectric Energy Harvesting Theory

Piezoelectric transducers can generate electricity under the traffic load based on the positive piezoelectric effect, which can be expressed by the constitutive equation consisting of mechanical parameters and electrical parameters. These piezoelectric units embedded in the pavement are mainly subjected to the vertical force, indicating a free mechanical boundary condition and open-circuit electrical boundary condition [30]. Here, the strain-voltage form is chosen for electromechanical conversion analysis, as shown in Equation (1) [23]:

(1) $$\{\begin{array}{l} S=s^{D}T+g^{t}D\\ E=-gT+\beta^{T}D \end{array}$$

where *S* is the strain, *S*^D^ is the elastic compliance constant, *T* is the stress, *g* is the voltage constant, *g*^t^ is the transposed *g*, *E* is the electrical field, *β*^T^ is the free dielectric isolation rate, and *D* is the electric displacement.

This paper chooses a stacked piezoelectric unit with a *d*_33_ mode (the electric field *E* and the stress *T* have the same direction) to obtain high energy conversion efficiency under a low-frequency state. Under the axial force, the open-circuit voltage *U*_oc_ and generated energy *W* of a piezoelectric unit can be calculated by the following Equations (2) and (3):

(2) $$U_{\mathrm{oc}}=-g_{33}T_{3}h$$

(3) $$W=\frac{1}{2}C_{p}U_{\mathrm{oc}}^{2}=\frac{1}{2}C_{p}g_{33}{}^{2}T_{3}{}^{2}h^{2}$$

where *d*_33_ is the piezoelectric coefficient, *h* is the thickness of the unit, and *C*_p_ is the internal equivalent capacitance.

When the piezoelectric unit is acted on by the sinusoidal load and connected with load resistance *R*_L_, then *D*, *T*, *E*, and S are sinusoidal functions of time *t* and have the same frequency [23]. The output voltage *U*_out_(*t*) and output power *P*_out_(*t*) can be described by Equations (4) and (5):

(4) $$U_{\mathrm{out}\;}(t)=\frac{U_{\mathrm{oc}}R_{L}}{R_{p}+R_{L}}=-g_{33}T_{0}h\frac{R_{L}}{R_{p}+R_{L}}\sin(\omega t)$$

(5) $$P_{\mathrm{out}\;}(t)=\frac{U_{\mathrm{out}\;}^{2}}{R_{L}}={\left[g_{33}T_{0}h\sin(\omega t)\right]}^{2}\frac{R_{L}}{{\left(R_{p}+R_{L}\right)}^{2}}$$

where *ω* is the angular velocity, *R*_p_ is the internal equivalent resistance, and *T*_0_ is the stress magnitude.

By integration of the load time and the voltage, the output electric energy *W*_out_ and the output power *P*_out_ under one sinusoidal excitation can be seen in Equations (6) and (7) [15]:

(6) $$W_{\mathrm{out}}=\frac{\pi }{\omega }{\left(g_{33}T_{0}h\right)}^{2}\frac{R_{L}}{{\left(R_{p}+R_{L}\right)}^{2}}$$

(7) $$P_{\mathrm{out}}=\frac{1}{2}{\left(g_{33}T_{0}h\right)}^{2}\frac{R_{L}}{{\left(R_{p}+R_{L}\right)}^{2}}$$

According to the equations above, it is clear that the electric energy is correlated with the material parameters and thickness of the piezoelectric unit and the traffic loads. The load resistance also has a significant influence on the energy generation, and the maximum output energy can be obtained at the matched impedance (*R*_L_ = *R*_p_) referring to [31]. Therefore, strategies such as piezoelectric material optimization, geometric design optimization, and impedance matching can be adopted to improve the energy conversion efficiency of the piezoelectric unit.

## 3. Development and Manufacture of the Piezoelectric Device

### 3.1. Development of Piezoelectric Unit

The material and structure design of the piezoelectric units determine the conversion efficiency and structure durability [32]. The lead zirconate titanate piezoelectric (PZT) ceramics, PZT-5H, was chosen because of its excellent piezoelectric properties with a high piezoelectric coefficient *d_33_*, a high electromechanical coupling factor *k_33_*, and a high compressive strength under the vertical load. The material parameters of PZT-5H are shown in Table 1.

**Figure 1:**
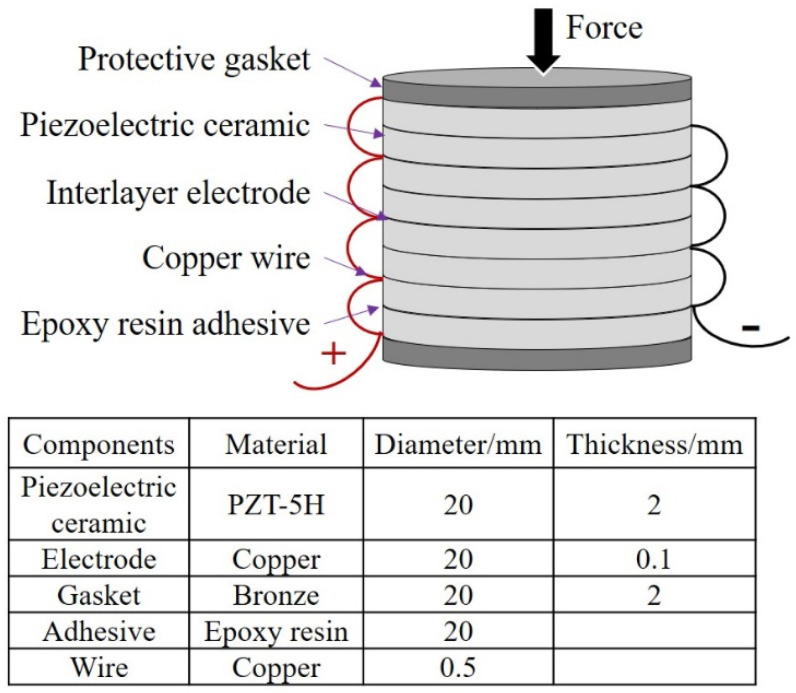
The structure of the stacked piezoelectric unit.

To improve the efficiency and fatigue life of piezoelectric transducers, researchers optimized the structure of the piezoelectric transducer to layer the bridge type [8], multilayer stack type [33], and multilayer cantilever type [34]. However, these structures have not been widely used due to complex preparation processes and high costs. Therefore, a stacked piezoelectric unit consisting of several piezoelectric ceramics, electrodes, epoxy resin adhesive, and protective gasket was proposed and fabricated referring to the author’s previous work [19]. Figure 1 illustrates the components and structural parameters of the piezoelectric unit, which is about 20.9 mm in height and formed with eight pieces of piezoelectric ceramics. Each of the piezoelectric ceramic has the same dimension of 20.0 mm in diameter and 2.0 mm in thickness. These piezoelectric ceramics are connected in parallel (as shown in Figure 1) to optimize the voltage and current properties. In addition, two 2.0 mm thick protective gaskets made of bronze are added on both sides. The ultra-thin epoxy resin acts as the interlayer adhesive to improve the integrity of the unit. The structural parameters and materials of the other components, such as interlayer electrodes and copper wire, can also be seen in Figure 1. The stacked piezoelectric units are industrially manufactured by Zhejiang Jiakang Electronics Co., Ltd., Zhejiang, China to avoid the influence of assembly accuracy and material variations on the piezoelectric performance.

### 3.2. Manufacture of the Piezoelectric Device

**Figure 2:**
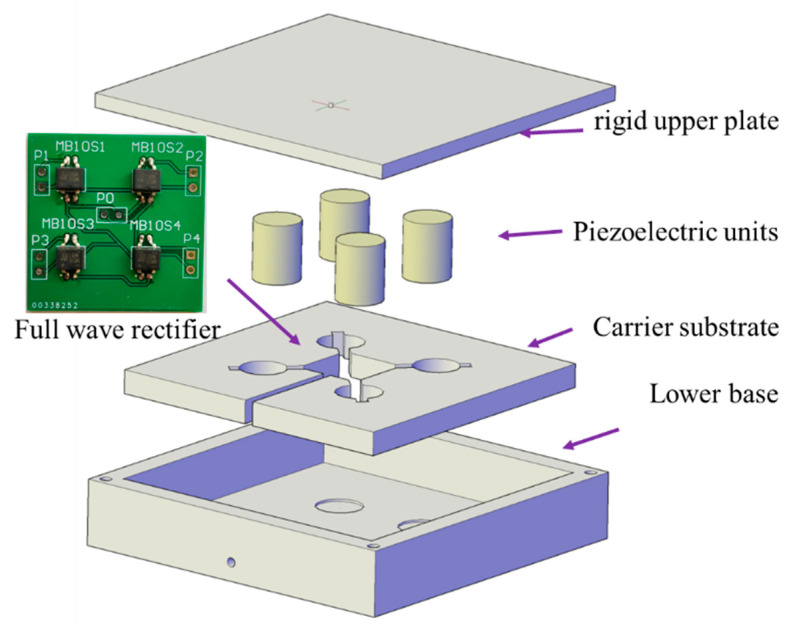
The main components of the stack units-based piezoelectric device.

The piezoelectric units and conditioning circuits can be damaged by the traffic load easily without the package structure when being installed in the pavement structure [17]. Additionally, package structures can transfer and amplify the vertical stress acting on the units through the upper plate. Moreover, it can provide coordinated deformation with the adjacent pavement structure. As shown in Figure 2, the stack piezoelectric transducer array consisting of four units was installed inside the device. All the piezoelectric units are connected to the full-wave rectifier and connected in parallel mode to reduce the adverse effects of uneven load and optimize the voltage and current properties under heavy vehicle load [9,15]. Beyond only these piezoelectric units and full-wave rectifier, a complete piezoelectric device also includes a high-strength shell, carrier substrate or positioning plate, and other components which are illustrated in Figure 2. In the load tests, the size of the piezoelectric device is determined to be 150 mm × 150 mm × 36 mm from the perspective of stress distribution, tire contact area, and production cost. The bearing shell is composed of the high-strength upper plate and the lower base with a gap design between the two components [21], and the two components are connected using the rubber sealings and bolts. The 150 mm long, 150 mm wide, 6 mm thick upper plate is made of an aluminum alloy with bolt holes and a fixing slot. These slots are reserved for the piezoelectric unit installation with a dimension of 20 cm in diameter and 1 mm in height. Additionally, the lower base and carrier substance were made of nylon 66 with the physical properties of light weight and high strength. As shown in Figure 2, the carrier substrate was placed between the upper plate and the lower base to fix piezoelectric units, rectifiers, and wires. It should be noted that the number of units and the size of the box-like device can be subsequently optimized according to the traffic volume and loads.

The manufacturing process is accomplished as follows. Firstly, by installing the carrier substrate in the lower base. Secondly, by fixing the piezoelectric units and rectifiers in the carrier substrate and using electronic adhesive to seal the printed circuit board (PCB) of the rectifiers (Figure 2). Thirdly, by placing the waterproof silicone gasket between the upper plate and the sidewall of the lower base. Fourthly, by installing and leveling the upper plate to make its fixing slots and units contact closely. Finally, by using silicone rubber sealing material to conduct the waterproof treatment.

## 4. Analysis on Factors Influencing Piezoelectric Properties

### 4.1. Effect of Stress Distribution

According to Equations (6) and (7), the output electrical energy is related to the vertical stress acting on these units. However, the vertical stress on each unit can fluctuate due to fabrication inaccuracy, geometric variations, and wheel wandering. This can cause varying electrical properties and difficulty in energy collection. Therefore, it is necessary to clarify the influence of stress distribution on power generation. Based on the theoretical analysis above, the output energy *W*_parallel_ and voltage *U*_parallel_ generated by n piles of piezoelectric units in parallel connection are shown in Equations (8) and (9):

(8) $$U_{\mathrm{parallel}}=\frac{1}{n}\sum_{i=1}^{n}U_{i}=\frac{1}{n}g_{33}h\sum_{i=1}^{n}T_{i}$$

(9) $$W_{\mathrm{parallel}}=\frac{1}{2}nC_{p}U_{\mathrm{parallel}}^{2}=\frac{1}{2n}C_{p}g_{33}{}^{2}h^{2}{\left(\sum_{i=1}^{n}T_{i}\right)}^{2}$$

The vertical stresses acting on these units are transferred from the tire load applied to the upper plate of the piezoelectric device, so that the total stress is constant. Based on Equation (9), the increase in the unit number will cause a decrease in the electrical energy under open-circuit conditions with the same unit and tire load. However, the stress distribution of units will not affect the generated energy when the unit array is connected in a parallel connection. To further investigate the influence of the nonuniform stress of the units on the output energy, the vertical compressive stresses on the selected two piezoelectric units are set as *T*_A_ and *T*_B_ respectively, and *T*_A_ ≥ *T*_B_. The electrical energy *W*_A_ and *W*_AIIB_ can be calculated in Equations (10) and (11):

(10) $$W_{A}=\frac{1}{2}c_{p}g_{33}{}^{2}h^{2}T_{A}^{2}$$

(11) $$W_{A\|B}=\frac{1}{4}c_{p}g_{33}{}^{2}h^{2}{\left(T_{A}+T_{B}\right)}^{2}$$

where *W*_A_ is the electrical energy generated by unit A and *W*_AIIB_ is the electrical energy generated by units A and B connected in parallel mode.

According to Yang’s analysis on the effect of nonuniform stress [15], *α* is introduced to describe the stress difference of the two units and *β* is adopted to illustrate the ratio of *W*_AIIB_ and *W*_A_:

(12) $$\alpha =\frac{T_{B}}{T_{A}}$$

(13) $$\beta =\frac{W_{A\|B}}{W_{A}}=\frac{1}{2}\frac{{\left(T_{A}+T_{B}\right)}^{2}}{T_{A}{}^{2}}=\frac{1}{2}{\left(1+\alpha \right)}^{2}$$

It can be seen from Equations (12) and (13) that the result of *β* has a positive quadratic relationship with the value of *α.* If *α* belongs to ($\sqrt{2}$−1, 1], which is under a small nonuniform stress state, the electrical energy generated by the units in parallel connection will be larger than that of a single unit. This indicates that (1) when the stress difference between piezoelectric units is large, e.g., *α* belongs to (0, $\sqrt{2}$−1], these piezoelectric units should be rectified and output individually [23] and (2) when the stress difference between piezoelectric units is small, e.g., *α* belongs to ($\sqrt{2}$−1, 1], these piezoelectric units should be rectified and connected in parallel connection to improve the energy conversion efficiency [15].

### 4.2. Effect of Load Impedance

**Figure 3:**
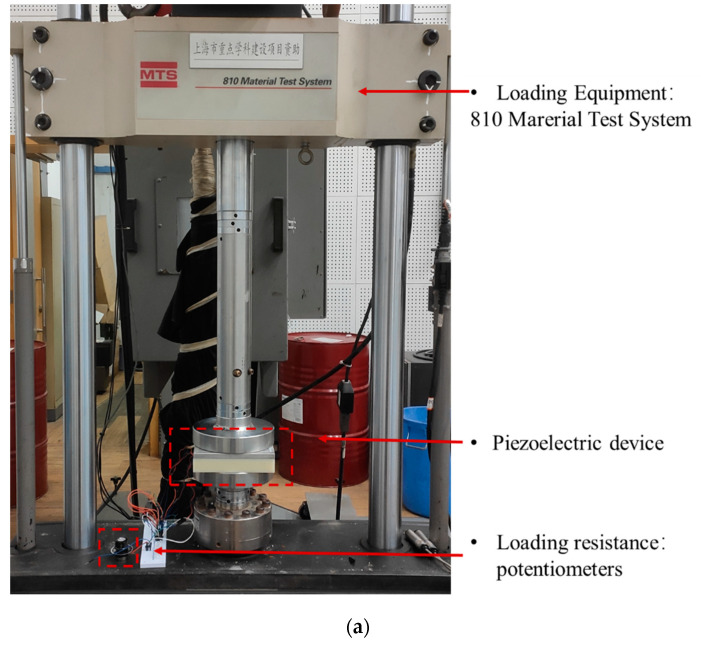

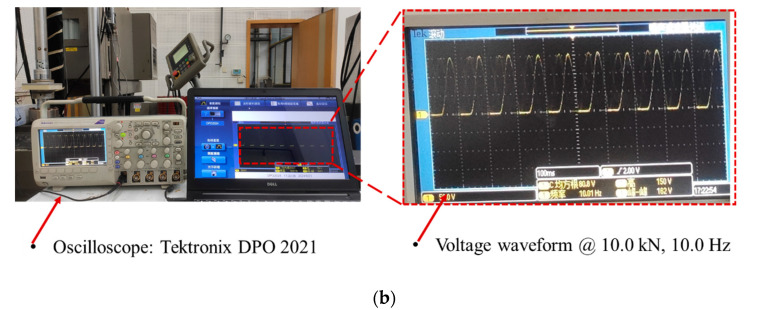
The mechanical testing and simulation system: (**a**) the loading test system; (**b**) the electrical properties monitoring system.

Equation (7) indicates that with the matched impedance, the developed piezoelectric device can obtain the maximum power generation. As a result, the mechanical testing and simulation system was performed in the laboratory to investigate the effect of load resistance on the electrical performance, as shown in Figure 3. The test system consisted of the loading simulation equipment and the loading resistance and electrical properties monitoring equipment. The loading simulation equipment used is the 810 Material Test System (MTS). It is able to provide loads with specific load frequency and load magnitude to simulate the vehicle load. Waveforms such as sinusoidal form, half-wave sinusoidal form, and haversine form can be chosen [35,36]. In this case, the vehicle load was simplified as a sinusoidal wave [22], which can be calculated by Equation (14):

(14) $$F(t)=F_{0}\sin(2\pi ft)+F_{m}$$

where *F*(*t*) is the function of the sin wave, *F*_0_ is the load magnitude, *f* is the load frequency, and *F_m_* is the mean load.

The electrical properties monitoring equipment used is the Tektronix DPO 2024 oscilloscope, which has the feature of four analog channels, 200.0 MHz bandwidth, and sample rates up to 1.0 GS/s. A high-voltage probe GENTEK G3100 was used to measure the voltage of the potentiometers connected with the rectifier circuit board. Then, the output power of the circuit was calculated by the integration of the response time and the measured voltage in the voltage waveform. The actual voltage waveform at 10.0 kN and 10.0 Hz can be seen in Figure 3 and the output power can be determined in Equation (15):

(15) $$P_{L}=\frac{W}{t}=\frac{\int_{0}^{\Delta t}\frac{U_{L}^{2}}{R_{L}}d(t)}{\Delta t}$$

where *P_L_* is the output power of the circuit, *U_L_* is the measured output voltage, and Δ*t* is the accumulated time of output voltage waveform.

**Figure 4:**
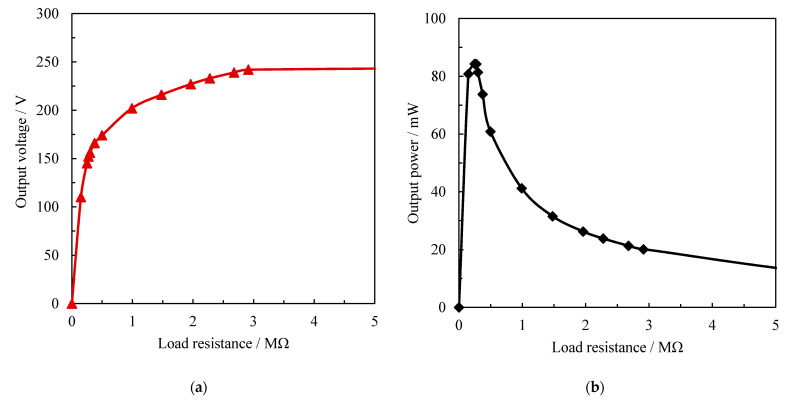
Electrical properties of the piezoelectric device with load impedance: (**a**) the output voltage, (**b**) the output power.

The magnitude and frequency of sinusoidal load were set as 10.0 kN and 10.0 Hz in the load test. The output voltage and power with different load resistance are shown in Figure 4. As can be seen from Figure 4, the output voltage of the piezoelectric device increased with the load impedance increase. When the resistance value was larger than 3.0 MΩ, the voltage was stable around 240 V and the circuit changed to an open-circuit state. In addition, the profile of output power presented a unimodal distribution. Under this testing condition, the maximum output power was 84.3 mW and loaded with a 275.0 kΩ resistance, which was enough to supply the low-power sensor [31]. Moreover, the increase of load impedance from this optimal resistance to 3.0 MΩ caused the output power to decrease significantly. This may be due to the reason that an increase in load resistance resulted in the output voltage increase, inducing the increase in output power accordingly. However, when the resistance value exceeded a specific value, the output voltage increased slowly and the output current decreases rapidly, ultimately resulting in a decrease in output power. As shown in the waveform of Figure 4, to obtain an excellent electrical performance of the piezoelectric device, the load impedance should be controlled to 100–1000 kΩ for the piezoelectric device developed in this paper.

### 4.3. Effect of Vehicle Load

The mechanical testing and simulation system was performed to further investigate the effects of load magnitude and speed on the piezoelectric performance. The load magnitudes applied by the MTS were set to 3.0 kN, 5.0 kN, 10.0 kN, 15.0 kN, 20.0 kN, and 25.0 kN at a fixed load frequency of 10.0 Hz. The load frequency was performed to simulate the load speed of vehicles, and were set from 2.0 Hz to 14.0 Hz with an interval of 2.0 Hz at the fixed load magnitude of 10.0 kN. The output voltage and power of the circuit were measured and calculated on all load conditions.

#### 4.3.1. Effect of Load Magnitude

**Figure 5:**
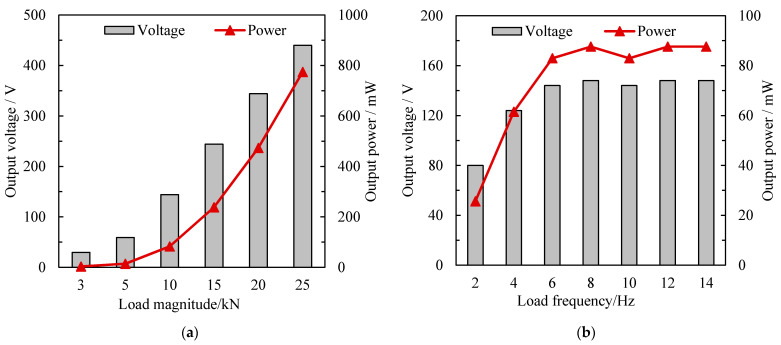
Electrical properties of the piezoelectric device with different load conditions: (**a**) effect of load magnitude at 10 Hz, (**b**) effect of load frequency at 10 kN.

The output voltage and power of the piezoelectric device from each load at 10.0 Hz are shown in Figure 5a. In Figure 5a, the output voltage increases linearly with the load magnitudes. However, the relationship between the output voltage and the applied load did not follow the rule of Equation (2). Without considering the unit of each parameter, the regression equation is determined by Equation (16) with a coefficient of determination of 0.9986:

*U*_L_ = 18.85 × *F*_i_ − 34.91 (16)

where *F*_i_ is the different load magnitude at 10.0 Hz.

Equation (16) indicated that when the vertical load acted on the device, the package structure will undertake the load as well, weakening the electrical properties of the device. Furthermore, the output power had a positive quadratic polynomial relationship with the load increases, which was consistent with the piezoelectric theory. The output power can be calculated by Equation (17) and the coefficient of determination is 0.9999:

*P*_L_ = 37.82 × *F*_i_^2^ − 110.79 × *F*_i_ + 78.60 (17)

As for a truck wheel load of 25.0 kN, the output voltage and power can reach 440.0 V and 774.4 mW, which shows that the proposed piezoelectric device can have high piezoelectric performance when subjected to heavy traffic loads.

#### 4.3.2. Effect of Load Frequency

With the load magnitude set at 10.0 kN, the measured voltages and calculated powers under each load frequency were shown in Figure 5b. As can be seen from Figure 5b, the output voltage and power increased gradually with the increase in load frequency at the initial stage, but the increasing tendency slowed down and fluctuate around 86.0 mW when the frequency was greater than 8.0 Hz. According to the field study of the actual vehicle load, the frequency of 8.0 Hz has the corresponding vehicle speed of 30.0 km/h. It indicated that the proposed piezoelectric device held a stable piezoelectric performance when installed in the roadways. More load conditions need to be conducted by indoor and field tests to verify the electrical properties in future studies.

### 4.4. Effect of Loading Times

**Figure 6:**
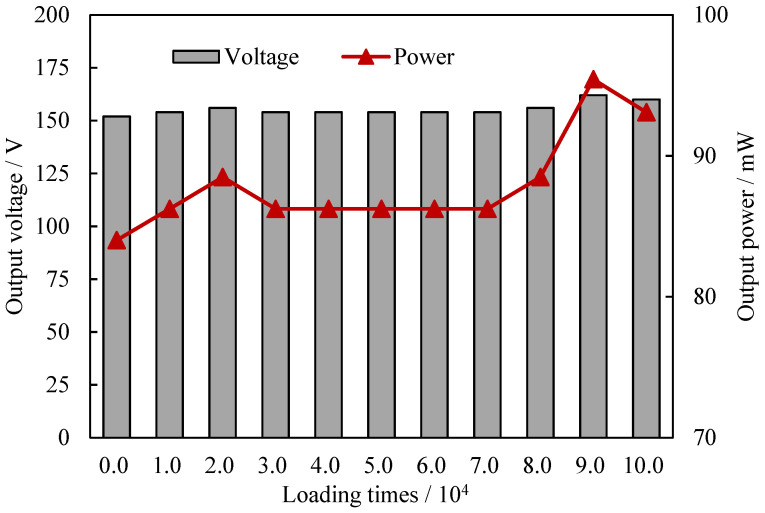
Electrical properties of the piezoelectric device with different loading times.

The fatigue test under dynamic loads was conducted to evaluate the electrical fatigue property of the developed piezoelectric device. The mechanical testing and simulation system was the same as that of Section 4.2. Here, a total of 100,000 cyclic loads were applied by the MTS and the sine load was set to 10.0 kN and 10.0 Hz. The voltage was measured by an oscilloscope every 10,000 loadings and the output voltage and power are shown in Figure 6. In Figure 6, during the whole loading process, the voltage and output power of the circuit varied slightly around 155 V and 86.0 mW, respectively. The difference between the minimum voltage and the maximum voltage was approximately 6.6%, while that of the output power was approximately 13.6%. However, no significant electrical attenuation appeared in the fatigue test, indicating a good electrical fatigue performance. The fatigue test indicated that the proposed device can meet the cyclic dynamic loading requirement for pavement energy harvesting.

## 5. On-Site Piezoelectric Properties Test

**Figure 7:**
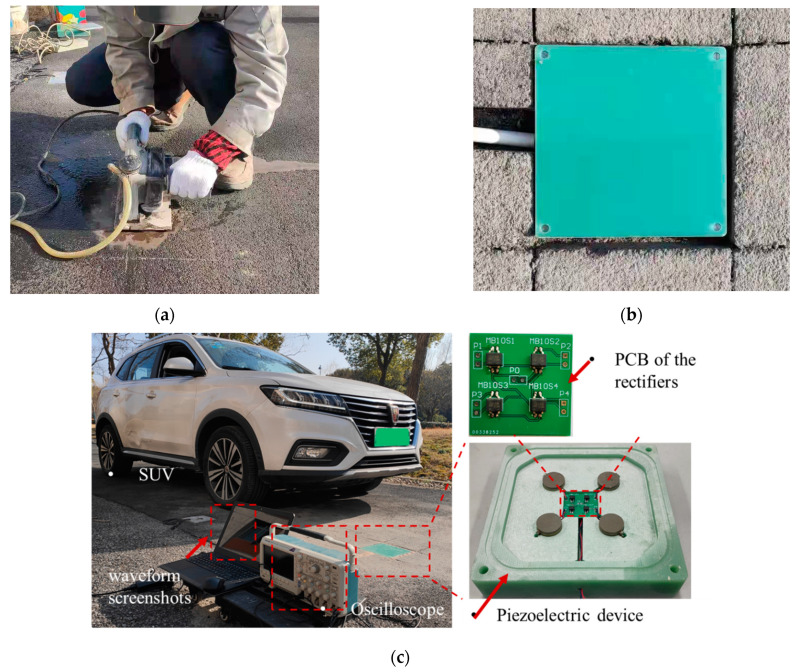
On-site tests of the piezoelectric device: (**a**) cutting the pavement surface, (**b**) installing the PEH, and (**c**) applying vehicle load.

**Figure 8:**
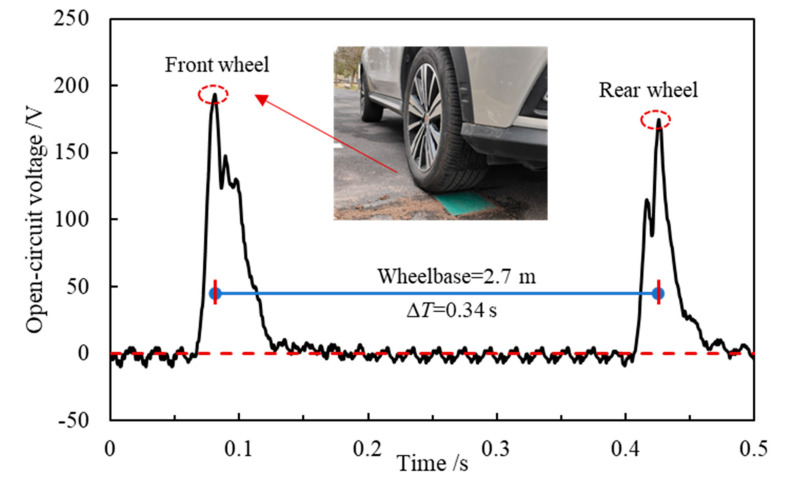
The open-circuit voltage under actual vehicle load.

As indicated by the analysis above, the proposed piezoelectric device has a novel electrical performance under the simulated vehicle load in the indoor test. The on-site performance evaluation was conducted in Tongji University to test the actual piezoelectric properties in real road conditions. As shown in Figure 7, the hole and slot were cut in the asphalt pavement surface using a road cutting machine for installing the piezoelectric device and wire. The height of the hole was larger than the thickness of the device so that the hole bottom can be flattened with cementitious mortar. After the installation of the device and wire, the interface between the device and surrounding pavement and the wire slot were treated with high-strength filling materials to avoid damaging the structural strength and service performance of the pavement [13,37]. Then, the oscilloscope was connected to the device to record the open-circuit voltage. During the on-site test, a sport utility vehicle (SUV) with an average single-wheel load of 5 kN was driven to applied loads on the surface of the upper plate of the piezoelectric device. The open-circuit voltage waveform with a sample interval of 1250 Hz is illustrated in Figure 8. In Figure 8, the waveform had two peak voltages around 190 V and 170 V respectively, indicating a high energy conversion efficiency. According to Section 4.3.1, the front wheel load was larger than that of the rear load when the tire fully acted on the device. Considering that the wheelbase is 2.7 m and the time difference ∆T between the two peak voltages is 0.34 s, the calculated load speed was about 28.6 km/h. It indicates this proposed piezoelectric device can also be used to detect vehicle speed, number of axles, axle loads, and vehicle classification [14,30]. The authors will conduct more research on the relationship between electrical properties and traffic data in the future.

## 6. Conclusions

The piezoelectric energy harvester for roadway application can alleviate energy shortages and environmental problems, and also has bright prospects for functional and intelligent roads. However, the development of this module is still in the exploratory stage. Its electrical performance needs to be investigated further to improve the power generation efficiency and compatibility in roadway applications. This paper proposed and fabricated a high-performance stack units-based piezoelectric device and tested the piezoelectric properties by indoor and field tests. The influences of stress distribution, load resistance, vehicle load, and loading times on the piezoelectric performance were analyzed. In addition, the on-site piezoelectric performance was also validated in the actual road conditions. The main conclusions are as follows:

(1) The unit number has a negative relationship with the electrical energy under open-circuit conditions, and the stress distribution of units does not influence the power generation when the unit array is in a parallel connection mode. However, the optimal connection mode was affected by the stress difference of units. The smaller the stress difference is, the higher the obtainable electrical performance of the piezoelectric units in parallel connection.

(2) The output power of the piezoelectric device with varying resistance has a unimodal distribution. It shows the circuit obtained a maximum power of 84.3 mW with 275.0 kΩ resistance at 10.0 kN and 10.0 Hz loading condition. The generated electricity is enough to supply the low-power sensor.

(3) The electrical performance of the piezoelectric device, loaded with the matched resistance, was positively correlated with the load magnitude and load frequency. The load tests indicate that the proposed piezoelectric device had a high and stable piezoelectric performance. Moreover, good electrical fatigue performance is also found under cyclic dynamic loadings.

(4) The novel piezoelectric properties of the developed piezoelectric device in real road conditions were confirmed by the field test, and the voltage waveform can be used to detect traffic parameters.

## Figures and Tables

**Table 1:** Material parameters of PZT-5H.

Material Properties		Value	Material Properties		Value
Piezoelectric charge constants (pC/N)	*d*_33_	750	Relative dielectric constants	*ε* ^T^_33,*r*_	4500
	*d*_31_	−320		*ε* ^T^_31,*r*_	4410
Piezoelectric voltage constants (10^−3^ Vm/N)	*g*_33_	19	Electro-mechanical coupling factor	*K*_33_	0.68
	*g*_31_	−8.2	Elastic Modulus (10^10^ N/m^2^)	*E*	6.1

## Data Availability

Not applicable.
